# Effects of Risperidone and Prenatal Poly I:C Exposure on GABA_A_ Receptors and AKT-GSK3β Pathway in the Ventral Tegmental Area of Female Juvenile Rats

**DOI:** 10.3390/biom12050732

**Published:** 2022-05-23

**Authors:** Shiyan Chen, Jiamei Lian, Yueqing Su, Chao Deng

**Affiliations:** 1 The First Affiliated Hospital of Fujian Medical University, Fuzhou 350005, China; cshiyan@163.com; 2 Antipsychotic Research Laboratory, Illawarra Health and Medical Research Institute, Wollongong, NSW 2522, Australia; jlian@uow.edu.au (J.L.); syq0506@126.com (Y.S.); 3 School of Medical, Indigenous and Health Sciences and Molecular Horizons, University of Wollongong, Wollongong, NSW 2522, Australia; 4 Fujian Maternity and Child Health Hospital, College of Clinical Medicine for Obstetrics & Gynaecology and Paediatrics, Fujian Medical University, Fuzhou 350001, China

**Keywords:** maternal immune activation, risperidone, ventral tegmental area, GSK3β, GABA_A_ receptor

## Abstract

The ventral tegmental area (VTA) in the ventral midbrain is the origin of the dopaminergic neurotransmission pathways. Although GABA_A_ receptors and AKT-GSK3β signaling are involved in the pathophysiology of mental disorders and are modulated by antipsychotics, an unmet task is to reveal the pathological changes in these biomarkers and antipsychotic modulations in the VTA. Using a juvenile polyriboinosinic-polyribocytidylic acid (Poly I:C) psychiatric rat model, this study investigated the effects of adolescent risperidone treatment on GABA_A_ receptors and AKT/GSK3β in the VTA. Pregnant female Sprague–Dawley rats were administered Poly I:C (5mg/kg; i.p) or saline at gestational day 15. Juvenile female offspring received risperidone (0.9 mg/kg, twice per day) or a vehicle from postnatal day 35 for 25 days. Poly I:C offspring had significantly decreased mRNA expression of GABA_A_ receptor β3 subunits and glutamic acid decarboxylase (GAD2) in the VTA, while risperidone partially reversed the decreased GAD2 expression. Prenatal Poly I:C exposure led to increased expression of AKT2 and GSK3β. Risperidone decreased GABA_A_ receptor β2/3, but increased AKT2 mRNA expression in the VTA of healthy rats. This study suggests that Poly I:C-elicited maternal immune activation and risperidone differentially modulate GABAergic neurotransmission and AKT-GSK3β signaling in the VTA of adolescent rats.

## 1. Introduction

Epidemiological and experimental evidence implicates gestational infections as one important factor in the pathogenesis of neuropsychiatric disorders. Maternal immune activation (MIA) during pregnancy increases the risk of the offspring developing neuropsychiatric disorders, such as schizophrenia, autism spectrum disorders and bipolar disorders, later in life [1,2,3,4]. Specific animal models of MIA, based upon the administration of immunogenic substances to the pregnant female, have been developed. The most commonly used approaches rely on mimicking maternal infection by treatment with the bacterial endotoxin lipopolysaccharide (LPS) and the double-stranded RNA (dsRNA) analog polyriboinosinic-polyribocytidylic acid (Poly I:C) [5,6,7,8]. Offspring with prenatal Poly I:C exposure may develop a spectrum of schizophrenia-like symptoms, including deficits in sensorimotor gating, working memory, latent inhibition and social interaction, and sensitivity to amphetamine [5,8]. Although sex differences have been observed in the rodent MIA models for schizophrenia [9,10], the majority of preclinical studies have been conducted in male offspring only, largely to avoid possible influence by estrogens [11]. In addition, our recent study found that prenatal Poly I:C challenge caused behavioral deficits in female adolescent offspring rats [12]. Therefore, this study focused on female adolescent Poly I:C rats. A number of previous studies demonstrated that prenatal Poly I:C exposure caused neurotransmission deficits in dopaminergic, serotoninergic (5-HT), γ-aminobutyric acid (GABA), and glutamatergic N-methyl-D-aspartate (NMDA) receptors in the prefrontal cortex, hippocampus, nucleus accumbens and caudate putamen [12,13,14]; however, little attention has been paid to the ventral midbrain.

The ventral tegmental area (VTA), located in the ventral midbrain, is an origin nucleus of mesolimbic dopamine neurotransmission that contains the cell bodies of dopamine neurons that project their axons to the cortex and nucleus accumbens. Abnormal neurotransmissions in the mesolimbic dopamine pathway contribute to the pathophysiology of schizophrenia, while the blockade of dopamine D2 receptor (D2R) activity in the mesolimbic pathway is the main mechanism of antipsychotic drug action [15,16,17]. Dopamine neurons in the VTA are modulated by GABAergic interneurons [15,18]. Therefore, the ventral midbrain may play a pivotal role in the antipsychotic treatment of schizophrenia; however, it has not been well studied [19].

The protein kinase B (AKT)-glycogen synthase kinase 3 beta (GSK3β) signaling pathway is a G-protein-independent pathway mediated by the D2R. Dopamine-associated neuropsychiatric illnesses, such as schizophrenia and bipolar disorder, seem to be characterized by impairments in the AKT/GSK3β pathway [20,21,22,23,24], while AKT/GSK3β-dependent signaling pathways are involved in the actions of antipsychotics [25,26,27,28,29,30,31,32,33].

Over the past decade, since approximately one fifth of children and adolescents have been diagnosed with mental illness, antipsychotic prescriptions (mostly off-label) have increased rapidly for juveniles, despite a lack of knowledge about the safety and efficacy of antipsychotics in the developing brain [34,35,36]. Risperidone is the most widely used antipsychotic drug (accounting for ~70% of total prescriptions) for treating various childhood mental disorders, including depression, bipolar disorder, autism, and childhood-onset schizophrenia [34,37,38,39,40,41,42]. Since children/adolescents are in a critical period of brain development, and may be more sensitive to the antipsychotics than adults [37], it is vital to further understand the pharmacological mechanisms of antipsychotics in children/adolescents. Therefore, this study investigated the effect of risperidone on the expression of D2R, GABAA receptor and AKT/GSK3β signaling in the VTA, using a female juvenile Poly I:C rat model.

## 2. Materials and Methods

### 2.1. Animals and Treatment

The methods for establishing a Poly I:C rat model were conducted as previously reported, which showed a phenotype with schizophrenia-like behavioral deficits in both adolescent and adult offspring [12,14]. Briefly, time-mated pregnant Sprague–Dawley rats (gestational day (GD) 8; Animal Resource Centre, Perth, Australia) were housed individually in Techniplast GR1800 double-decker rat ventilated cages (IVCs) and allowed to habituate to their surroundings for one week. At GD 15, dams were injected with either Poly I:C (5 mg/kg dissolved in 0.2 mL 1% phosphate buffer saline, IP; *n* = 7; InvivoGen, Toulouse, France) or an equivalent volume of saline (*n* = 7). After postnatal day (PD) 21, female offspring rats were weaned and housed in Techniplast GR1800 double-decker rat ventilated cages with a divider, under environmentally controlled conditions (22 °C; light cycle from 07:00 to 19:00 and dark from 19:00 to 07:00) with ad libitum access to food and water. Each cage housed 2 rats from the same treatment group, and the divider (with perforated holes to allow the two rats to see, hear, and smell each other) separated the cage into two chambers of equal size, each with its own enrichment devices, including a plastic tunnel, a wood stick, and nesting materials with corncob bedding. Rats were administered risperidone (0.9 mg/kg mixed with cookie dough, twice per day; *n* = 6/group, Janssen Australia) or a vehicle (*n* = 6/group) orally from PD35 for 25 days, following the methods routinely used in our laboratory [43,44]. The final treatment was delivered 2 h prior to euthanasia. The rats were then euthanized by isoflurane anesthesia and decapitated, and the collected brains were frozen in liquid nitrogen and stored at −80 °C.

### 2.2. Brain Dissection

The discrete brain regions were collected using a brain microdissection puncture technique in a cryostat (at −10.5 °C ± 1.5 °C) as previously reported [31,32,45]. According to the brain atlas [46], the brain tissues through the VTA (Bregma −5.40 to −6.30 mm) were dissected and kept at −80 °C for future use.

### 2.3. RNA Isolation and Gene Expression Analysis by Real-Time qPCR

Total RNA from the VTA brain tissue was prepared using the PureLink RNA Mini Kit (#12183025; Invitrogen Life Technologies, Carlsbad, CA, USA). cDNA was synthesized from purified RNA using the High-Capacity cDNA Reverse Transcription Kits (#4368814; Thermo Fisher Scientific, Waltham, MA, USA). qRT-PCR was performed in duplicate on a Quant Studio™ qRT-PCR system (ThermoFisher, Waltham, MA, USA) using TaqMan^®^ Gene Expression Assays (Life Technologies, Sydney, NSW, Australia) for Drd2 (Rn00561126_m1), Gabrb1 (Rn00564146_m1), Gabrb2 (Rn00564149_m1), Gabrb3 (Rn00567029_m1), Gad1 (Rn00690300_m1), Gad2 (Rn00561244_m1), β-actin (Hs01060665_g1) and Gapdh (Rn01775763_g1), or SYBR™ Green PCR Master Mix (Life Technologies, Sydney, NSW, Australia) for Akt1 (forward primer: ggggaatatattaaaacctggc, reverse primer: gtcttcatcagctgacattg), Akt2 (forward primer: gagtcctacagaataccagg, reverse primer: aatctctgcaccataaaagc), Akt3 (forward primer: aaaggatccaaataaacgcc, reverse primer: aaggaggtacaagctttttg) and Gsk3b (forward primer: cactcaagaactgtcaagtaac, reverse primer: tccagcattagtatctgagg). The cycling parameters were 95 °C for 10 min followed by 40 cycles (95 °C for 15 s, 60 °C for 1 min). Target gene relative expression levels were normalized to two housekeeping genes, β-Actin and Gapdh. The 2−ΔΔCT method was used to calculate the results.

### 2.4. Statistical Analysis

SPSS software (version 21.0, IBM, Armonk, NY, USA) was used to analyze all data. The outliers were identified and removed using Boxplot. The Shapiro–Wilk test was used to examine the data distribution. Data were analyzed by two-way ANOVA (Poly I:C × risperidone). Post hoc Dunnett t-tests were followed for comparison between groups. All data are expressed as mean ± SEM, and statistical significance will be accepted when *p* < 0.05.

## 3. Results

### 3.1. Effects on the GABAergic Markers

**Figure 1:**
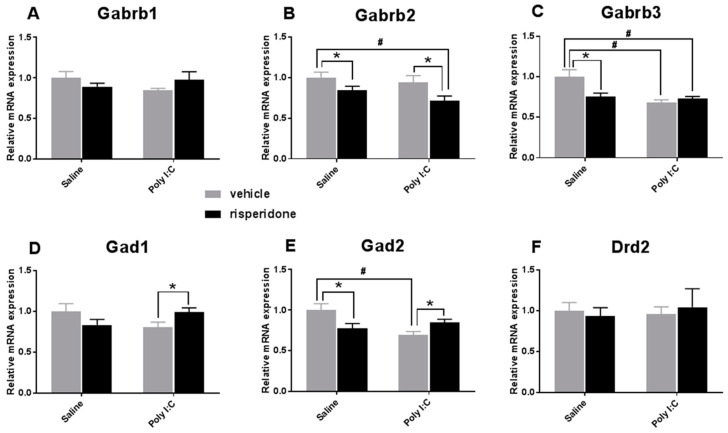
The effect of prenatal Poly I:C exposure and adolescent risperidone treatment on the mRNA expression of (**A**) GABA_A_ receptor β1(Gabrb1) subunit, (**B**) GABA_A_ receptor β2 (Gabrb2) subunit, (**C**) GABA_A_ receptor β3 (Gabrb3) subunit, (**D**) glutamic acid decarboxylase GAD1, (**E**) glutamic acid decarboxylase GAD2, and (**F**) dopamine D2 receptor in the VTA of female rats. Data were presented as mean ± SEM (*n* = 6/group). * *p* < 0.05, # *p* < 0.01.

The two-way ANOVA showed a significant main effect of risperidone (F_1, 20_ = 8.664, *p* = 0.008), but no effect of Poly I:C on Gabrb2 mRNA levels (F_1, 20_ = 2.132, *p* = 0.160); there were also no significant interactions between the two factors (F_1, 20_ = 0.319, *p* = 0.579). Adolescent risperidone treatment significantly decreased Gabrb2 expression in offspring rats with both prenatal Poly I:C and saline exposure (saline–risperidone vs. saline–vehicle, *p* = 0.045; Poly I:C–risperidone vs. Poly I:C–vehicle, *p* = 0.024) (Figure 1B).

There was a significant main effect of Poly I:C factor (F_1, 20_ = 10.24, *p* = 0.005) and a significant interaction between Poly I:C and risperidone factors (F_1, 20_ = 7.568, *p* = 0.012) on Gabrb3 expression, but without a significant effect of the risperidone factor (F_1, 20_ = 3.601, *p* = 0.072). Post hoc comparisons showed a more significant decrease in Gabrb3 mRNA levels in Poly I:C–vehicle offspring than in saline–vehicle rats (*p* = 0.003; Figure 1C). The saline–risperidone group also had lower Gabrb3 expression than the saline–vehicle group (*p* = 0.015), while there was no significant difference between the Poly I:C–vehicle and Poly I:C–risperidone groups (*p* > 0.05; Figure 1C). However, as shown in Figure 1A, both prenatal Poly I:C exposure and adolescent risperidone treatment had no significant effects on Gabrb1 mRNA expression (all *p* > 0.05).

Although there were no significant main effects of prenatal Poly I:C exposure (F_1, 20_ = 0.063, *p* = 0.804) or risperidone factor (F_1, 20_ = 0.015, *p* = 0.904) on Gad1 expression, there was a significant interaction between the two factors (F_1, 20_ = 6.265, *p* = 0.021). Poly I:C–vehicle rats had lower expression of Gad1 mRNA than the saline–vehicle group, but this difference was not significant (*p* = 0.241), while risperidone treatment increased Gad1 mRNA levels in Poly I:C offspring (Poly I:C–risperidonevs vs. Poly I:C–vehicle, *p* = 0.022; Figure 1D).

There was a main effect of prenatal Poly I:C exposure (F_1, 20_ = 4.237, *p* = 0.053) and a significant interaction between Poly I:C and risperidone on Gad2 expression (F_1, 20_ = 10.520, *p* = 0.004), although there was not a main effect of risperidone (F_1, 20_ = 0.341, *p* = 0.566). Post hoc comparisons showed that prenatal Poly I:C exposure significantly decreased Gad2 mRNA levels (Poly I:C–vehicle vs. saline–vehicle, *p* = 0.004), while risperidone treatment partially reversed this decrease in Poly I:C offspring (Poly I:C–risperidone vs. Poly I:C–vehicle, *p* = 0.015; Figure 1E). Interestingly, risperidone significantly reduced Gad2 mRNA levels in offspring with prenatal saline exposure (saline–risperidone vs. saline–vehicle, *p* = 0.024) (Figure 1E).

There was no significant effect of Poly I:C (F_1, 20_ = 0.060, *p* = 0.809) or risperidone (F_1,20_ = 0.005, *p* = 0.943) on D2R mRNA expression, and no significant interaction between the two factors (F_1, 20_ = 0.276, *p* = 0.605; Figure 1F).

### 3.2. Effects on Akt-GSK3β Signaling Pathway

**Figure 2:**
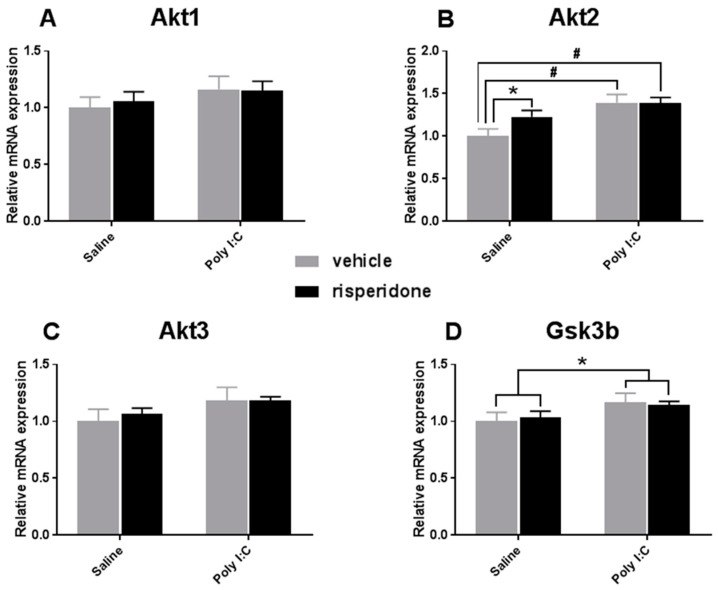
The effect of prenatal Poly I:C exposure and adolescent risperidone treatment on the mRNA expression of (**A**) Akt1, (**B**) Akt2, (**C**) Akt 3 and (**D**) GSK3β in the VTA of female rats. Data were presented as mean ± SEM (*n* = 6/group). * *p* < 0.05, # *p* < 0.01.

There was a significant main effect of Poly I:C on the expression of Akt2 (F_1, 20_ = 11.4, *p* = 0.003); however, there were no significant main effects of risperidone (F_1, 20_ = 1.782, *p* = 0.197), and also no significant interactions between the two factors (F_1, 20_ = 2.022, *p* = 0.170). Post hoc tests showed that the Akt2 mRNA level was significantly increased in Poly I:C offspring (Poly I:C–vehicle vs. saline–vehicle, *p* = 0.006; Poly I:C–risperidone vs. saline–vehicle, *p* = 0.003), while there was no significant difference between Poly I:C–risperidone and Poly I:C–vehicle (*p* > 0.05). Risperidone significantly increased Akt2 mRNA levels in the VTA of offspring rats with prenatal saline exposure (saline–risperidone vs. saline–vehicle, *p* = 0.039) (Figure 2B). No significant differences in Akt1 and Akt3 mRNA levels were observed in the VTA of Poly I:C- or risperidone-treated rats (Figure 2A,C). There was an overall effect of prenatal Poly I:C exposure with increased Gsk3β expression (F_1, 20_ = 4.355, *p* = 0.049); however, risperidone did not have any effects (F_1, 20_ = 0.004, *p* = 0.950) (Figure 2D).

## 4. Discussion

A number of studies have reported that maternal immune activation, such as prenatal Poly I:C exposure, causes deficits in various neurotransmitters and related cellular signaling pathways, particularly in the prefrontal cortex, hippocampus, nucleus accumbens and caudate putamen in rodent models [12,13,47]. This is the first study, however, to examine the effects of prenatal Poly I:C exposure and adolescent antipsychotic treatment on the expression of both GABAergic neurotransmission markers and AKT/GSK3β signaling in the VTA of adolescent Poly I:C rodent models.

Deficits in GABAergic neurotransmission have been implicated in the pathophysiology of schizophrenia [15,48,49]. Previous studies have reported that prenatal Poly I:C exposure caused abnormal expression of GABA_A_ receptor subunits in the cortex and hippocampus of rodent brains [13]. For example, prenatal Poly I:C exposure increased mRNA expression of GABA_A_ receptor α2/α4 subunits in the prefrontal cortex of juvenile offspring and α1/α2 subunits in the hippocampus of adult offspring, but decreased mRNA expression of the GABA_A_ receptor β3 (*Gabrb3*) subunit in the prefrontal cortex and β1 (*Gabrb1*) subunit in the hippocampus [12,47,50]. This study extended these findings by observing that prenatal Poly I:C exposure decreased *Gabrb3* mRNA expression in the VTA. There are two primary GABA-synthesizing enzymes: glutamate acid decarboxylase 67 (GAD67, also called GAD1) and glutamate acid decarboxylase 65 (GAD65, also called GAD2). GAD1 is the rate-limiting enzyme responsible for approximately 90% of GABA synthesis, while GAD2 is localized to the synaptic terminal and is largely involved in the regulation of postsynaptic GABA_A_ receptors [51]. Consistent with the changes in GABA_A_ receptors, this study revealed significantly decreased expression of GAD2 mRNA in the VTA of Poly I:C offspring rats. Previous studies have reported that GAD1 mRNA expression was reduced in the dorsal hippocampus of Poly I:C offspring mice, which could be revised by chronic lurasidone treatment [52].

This study further showed that chronic treatment with risperidone could reverse the decrease in *GAD2* expression in the VTA of Poly I:C offspring. It is unexpected that risperidone decreased mRNA expression of *Gad2* and GABA_A_ receptor *Gabrb2*/*Gabrb3* subunits in the VTA of healthy rats. Since GAD2 is a GABA-synthesizing enzyme, this finding is consistent with previous reports that acute risperidone treatment caused a significant reduction in extracellular GABA levels in the globus pallidus of rats [53], and that adolescent olanzapine treatment caused a long-term reduction in GABA levels in the nucleus accumbens of adult rats [54]. However, it is different from the finding, in the nucleus accumbens of healthy rats, that the expression of the GABA_A_ receptor *Gabrb1* subunit was elevated by 1-week treatment with aripiprazole and haloperidol, which is modulated by the PKA signaling pathway [55].

The Akt-GSK3β signaling pathway has been implicated in the pathophysiology of schizophrenia [29,56]. AKT has three isoforms, AKT1, AKT2, and AKT3, which play roles in a variety of processes, such as brain development and metabolism [57]. It has been repeatedly reported that there is decreased expression of AKT1 mRNA and protein levels in the prefrontal cortex and hippocampus in patients with schizophrenia [29,56]. AKT1 down-stream targets, such as GSK3β, are also altered in schizophrenia, including a decreased level of GSK-3β protein phosphorylation and GSK-3β mRNA in the prefrontal cortex [29,58]. A deficit in AKT1-GSK-3β signaling was also observed in the prefrontal cortex of Poly I:C offspring mice [59,60]. However, to date, while the majority of studies related to schizophrenia have focused on AKT1, particularly in the prefrontal cortex and hippocampus, this does not exclude the possible role of other AKT isoforms in the pathophysiology of schizophrenia [29]. This is the first study to investigate alterations in AKT-GSK3β signaling in the VTA of a schizophrenic animal model. We found that prenatal Poly I:C challenge led to increased mRNA expression of AKT2 and GSK3β; however, further study is important to examine whether AKT2 protein levels and GSK3β protein phosphorylation also have similar alterations in the VTA of Poly I:C rats. It is interesting that prenatal Poly I:C exposure had different effects on the expression of AKT isoforms in the midbrain nucleus (VTA) in this study and the prefrontal cortex/hippocampus in previous reports [59,60], suggesting a possible brain region-specific effect with respect to the influence of prenatal Poly I:C exposure on AKT signaling. Although the mechanisms underlying the brain regional differences are not clear, one possible explanation is that the various brain regions have differential neuroinflammation responses to maternal immune activation elicited by Poly I:C that have been reported previously [12,13]. Alternatively, the experimental differences should also be considered, such as age and species differences (adolescent rats in this study vs. adult mice in previous reports), or Poly I:C exposure time (GD15 in this study vs. GD9 in Willi et al. 2013 [60] or GD17 in Bitanihirwe et al. 2010 [61]), although the same Poly I:C dosage (5 mg/kg) was used in all of these studies. This study also found that adolescent risperidone treatment increased expression of Akt2 mRNA in the VTA of healthy rats, which is consistent with previous reports that various antipsychotics have been shown to increase the expression of Akt [25,28,32].

## 5. Conclusions

In summary, this study revealed the effects of prenatal Poly I:C-elicited immune activation and adolescent risperidone treatment on GABAergic neurotransmission markers and AKT/GSK3β signaling in the VTA of female rats. The results have shown that adolescent risperidone treatment is able to partly restore Poly I:C-induced alterations in the expression of GABAergic biomarkers. However, the mechanisms underlying these effects still have not been clearly revealed. Since recent reports showed that both prenatal Poly I:C-induced immune activation and antipsychotic treatment in rodents can induce long-lasting epigenetic modifications at multiple gene promoters [62,63,64], it is important to investigate the potential epigenetic mechanism underlying the GABAergic neurotransmission and AKT/GSK3β signaling changes triggered by prenatal immune activation and/or antipsychotic treatment. One limitation is that, due to the small sample of the VTA nucleus, only mRNA expression was examined in this study. Further studies are necessary to examine the protein levels with Western blot and GABAergic neurotransmission by electrophysiology recordings to fully reveal the effects of prenatal Poly I:C exposure and risperidone treatment. The other limitation is that only female juvenile rats have been examined in this study. Since sex differences have been observed in rodent Poly I:C schizophrenic models [9,10], further studies are necessary to investigate the effects of prenatal Poly I:C exposure and adolescent risperidone in the VTA of male rats.

## Data Availability

The data sets used and analyzed in this study are available from the corresponding authors on request.

## References

[1] Brown A.S., Susser E.S. (2002). In utero infection and adult schizophrenia. Ment. Retard. Dev. Disabil. Res. Rev..

[2] Brown A.S., Derkits E.J. (2010). Prenatal infection and schizophrenia: A review of epidemiologic and translational studies. Am. J. Psychiatry.

[3] Buka S.L., Cannon T.D., Torrey E.F., Yolken R.H. (2008). Maternal exposure to herpes simplex virus and risk of psychosis among adult offspring. Biol. Psychiatry.

[4] Khandaker G.M., Zimbron J., Lewis G., Jones P. (2013). Prenatal maternal infection, neurodevelopment and adult schizophrenia: A systematic review of population-based studies. Psychol. Med..

[5] Haddad F.L., Patel S.V., Schmid S. (2020). Maternal Immune Activation by Poly I:C as a preclinical Model for Neurodevelopmental Disorders: A focus on Autism and Schizophrenia. Neurosci. Biobehav. Rev..

[6] Meyer U. (2014). Prenatal poly(i:C) exposure and other developmental immune activation models in rodent systems. Biol. Psychiatry.

[7] Murray K.N., Edye M.E., Manca M., Vernon A.C., Oladipo J.M., Fasolino V., Harte M., Mason V., Grayson B., McHugh P.C. (2018). Evolution of a maternal immune activation (mIA) model in rats: Early developmental effects. Brain Behav. Immun..

[8] Reisinger S., Khan D., Kong E., Berger A., Pollak A., Pollak D. (2015). The poly(I:C)-induced maternal immune activation model in preclinical neuropsychiatric drug discovery. Pharmacol. Ther..

[9] Gogos A., Sbisa A., Witkamp D., Buuse M.V.D. (2020). Sex differences in the effect of maternal immune activation on cognitive and psychosis-like behaviour in Long Evans rats. Eur. J. Neurosci..

[10] Kokras N., Dalla C. (2014). Sex differences in animal models of psychiatric disorders. Br. J. Pharmacol..

[11] Prendergast B.J., Onishi K.G., Zucker I. (2014). Female mice liberated for inclusion in neuroscience and biomedical research. Neurosci. Biobehav. Rev..

[12] Su Y., Lian J., Hodgson J., Zhang W., Deng C. (2022). Prenatal Poly I:C Challenge Affects Behaviors and Neurotransmission via Elevated Neuroinflammation Responses in Female Juvenile Rats. Int. J. Neuropsychopharmacol..

[13] Bergdolt L., Dunaevsky A. (2019). Brain changes in a maternal immune activation model of neurodevelopmental brain disorders. Prog. Neurobiol..

[14] Osborne A.L., Solowij N., Babic I., Lum J.S., Newell K., Huang X.-F., Weston-Green K. (2019). Effect of cannabidiol on endocannabinoid, glutamatergic and GABAergic signalling markers in male offspring of a maternal immune activation (poly I:C) model relevant to schizophrenia. Prog. Neuro-Psychopharmacol. Biol. Psychiatry.

[15] Deng C., Pan B., Engel M., Huang X.-F. (2013). Neuregulin-1 signalling and antipsychotic treatment. Psychopharmacology.

[16] Ginovart N., Kapur S. (2012). Role of dopamine D(2) receptors for antipsychotic activity. Curr. Antipsychotics.

[17] Howes O., Kapur S. (2009). The dopamine hypothesis of schizophrenia: Version III--the final common pathway. Schizophr. Bull..

[18] Morikawa H., Paladini C. (2011). Dynamic regulation of midbrain dopamine neuron activity: Intrinsic, synaptic, and plasticity mechanisms. Neuroscience.

[19] Pan B., Deng C. (2019). Modulation by chronic antipsychotic administration of PKA- and GSK3β-mediated pathways and the NMDA receptor in rat ventral midbrain. Psychopharmacology.

[20] Benedetti F., Poletti S., Radaelli D., Bernasconi A., Cavallaro R., Falini A., Lorenzi C., Pirovano A., Dallaspezia S., Locatelli C. (2010). Temporal lobe grey matter volume in schizophrenia is associated with a genetic polymorphism influencing glycogen synthase kinase 3-β activity. Genes Brain Behav..

[21] Benedetti F., Bollettini I., Barberi I., Radaelli D., Poletti S., Locatelli C., Pirovano A., Lorenzi C., Falini A., Colombo C. (2013). Lithium and GSK3-β promoter gene variants influence white matter microstructure in bipolar disorder. Neuropsychopharmacology.

[22] Freyberg Z., Ferrando S.J., Javitch J. (2010). Roles of the Akt/GSK-3 and Wnt signaling pathways in schizophrenia and antipsychotic drug action. Am. J. Psychiatry.

[23] Gould T.D., Manji H.K. (2005). Glycogen synthase kinase-3: A putative molecular target for lithium mimetic drugs. Neuropsychopharmacology.

[24] Karege F., Méary A., Perroud N., Jamain S., Leboyer M., Ballmann E., Fernandez R., Malafosse A., Schürhoff F. (2012). Genetic overlap between schizophrenia and bipolar disorder: A study with AKT1 gene variants and clinical phenotypes. Schizophr. Res..

[25] Alimohamad H., Sutton L., Mouyal J., Rajakumar N., Rushlow W.J. (2005). The effects of antipsychotics on β-catenin, glycogen synthase kinase-3 and dishevelled in the ventral midbrain of rats. J. Neurochem..

[26] Alimohamad H., Rajakumar N., Seah Y.-H., Rushlow W. (2005). Antipsychotics alter the protein expression levels of β-catenin and GSK-3 in the rat medial prefrontal cortex and striatum. Biol. Psychiatry.

[27] Allen J.A., Yost J.M., Setola V., Chen X., Sassano M.F., Chen M., Peterson S., Yadav P.N., Huang X.-P., Feng B. (2011). Discovery of β-Arrestin—Biased dopamine D2 ligands for probing signal transduction pathways essential for antipsychotic efficacy. Proc. Natl. Acad. Sci. USA.

[28] Beaulieu J.-M., Gainetdinov R.R., Caron M.G. (2009). Akt/GSK3 signaling in the action of psychotropic drugs. Annu. Rev. Pharmacol. Toxicol..

[29] Emamian E.S. (2012). AKT/GSK3 signaling pathway and schizophrenia. Front. Mol. Neurosci..

[30] Li X., Rosborough K.M., Friedman A.B., Zhu W., Roth K. (2007). Regulation of mouse brain glycogen synthase kinase-3 by atypical antipsychotics. Int. J. Neuropsychopharmacol..

[31] Pan B., Huang X.F., Deng C. (2016). Chronic administration of aripiprazole activates GSK3β-dependent signalling pathways and up-regulates GABAA receptor expression and CREB1 activity in rats. Sci. Rep..

[32] Pan B., Huang X.-F., Deng C. (2016). Aripiprazole and Haloperidol Activate GSK3β-Dependent Signalling Pathway Differentially in Various Brain Regions of Rats. Int. J. Mol. Sci..

[33] Sutton L.P., Rushlow W.J. (2012). The dopamine D2 receptor regulates Akt and GSK-3 via Dvl-3. Int. J. Neuropsychopharmacol..

[34] Karanges E.A., Stephenson C.P., McGregor I.S. (2014). Longitudinal trends in the dispensing of psychotropic medications in Australia from 2009–2012: Focus on children, adolescents and prescriber specialty. Aust. N. Z. J. Psychiatry.

[35] Memarzia J., Tracy D., Giaroli G. (2014). The use of antipsychotics in preschoolers: A veto or a sensible last option?. J. Psychopharmacol..

[36] Olfson M., Blanco C., Wang S., Laje G., Correll C.U. (2014). National trends in the mental health care of children, adolescents, and adults by office-based physicians. JAMA Psychiatry.

[37] Caccia S. (2013). Safety and pharmacokinetics of atypical antipsychotics in children and adolescents. Paediatr. Drugs.

[38] Fraguas D., Correll C.U., Merchán-Naranjo J., Rapado-Castro M., Parellada M., Moreno C., Arango C. (2011). Efficacy and safety of second-generation antipsychotics in children and adolescents with psychotic and bipolar spectrum disorders: Comprehensive review of prospective head-to-head and placebo-controlled comparisons. Eur. Neuropsychopharmacol..

[39] Hoekstra P.J. (2014). Risperidone for non-psychotic disorders in paediatric patients: Which child is to benefit?. Dev. Med. Child Neurol..

[40] Olfson M., Crystal S., Huang C., Gerhard T. (2010). Trends in antipsychotic drug use by very young, privately insured children. J. Am. Acad. Child Adolesc. Psychiatry.

[41] Seida J.C., Schouten J.R., Boylan K., Newton A.S., Mousavi S.S., Beaith A., Vandermeer B., Dryden D.M., Carrey N. (2012). Antipsychotics for children and young adults: A comparative effectiveness review. Pediatrics.

[42] Sharma A., Shaw S.R. (2012). Efficacy of risperidone in managing maladaptive behaviors for children with autistic spectrum disorder: A meta-analysis. J. Pediatr. Health Care.

[43] De Santis M., Lian J., Huang X.-F., Deng C. (2016). Early Antipsychotic Treatment in Juvenile Rats Elicits Long-Term Alterations to the Dopamine Neurotransmitter System. Int. J. Mol. Sci..

[44] Pan B., Lian J., Deng C. (2018). Chronic antipsychotic treatment differentially modulates protein kinase A- and glycogen synthase kinase 3 beta-dependent signaling pathways, N-methyl-D-aspartate receptor and γ-aminobutyric acid A receptors in nucleus accumbens of juvenile rats. J. Psychopharmacol..

[45] Pan B., Chen J., Lian J., Huang X.-F., Deng C. (2015). Unique Effects of Acute Aripiprazole Treatment on the Dopamine D2 Receptor Downstream cAMP-PKA and Akt-GSK3β Signalling Pathways in Rats. PLoS ONE.

[46] Paxinos G., Watson C. (2007). The Rat Brain in Sterotaxic Coordinates.

[47] Richetto J., Calabrese F., Riva M.A., Meyer U. (2014). Prenatal immune activation induces maturation-dependent alterations in the prefrontal GABAergic transcriptome. Schizophr. Bull..

[48] De Jonge J.C., Vinkers C.H., Pol H.E.H., Marsman A. (2017). GABAergic Mechanisms in Schizophrenia: Linking Postmortem and In Vivo Studies. Front. Psychiatry.

[49] Hoftman G.D., Volk D.W., Bazmi H.H., Li S., Sampson A.R., Lewis D.A. (2015). Altered cortical expression of GABA-related genes in schizophrenia: Illness progression vs developmental disturbance. Schizophr. Bull..

[50] Richetto J., Labouesse M., Poe M.M., Cook J.M., Grace A.A., Riva M.A., Meyer U. (2015). Behavioral Effects of the Benzodiazepine-Positive Allosteric Modulator SH-053-2′F-S-CH3 in an Immune-Mediated Neurodevelopmental Disruption Model. Int. J. Neuropsychopharmacol..

[51] Lazarus M.S., Krishnan K., Huang Z.J. (2015). GAD67 deficiency in parvalbumin interneurons produces deficits in inhibitory transmission and network disinhibition in mouse prefrontal cortex. Cereb. Cortex.

[52] Luoni A., Richetto J., Longo L., Riva M. (2017). Chronic lurasidone treatment normalizes GABAergic marker alterations in the dorsal hippocampus of mice exposed to prenatal immune activation. Eur. Neuropsychopharmacol..

[53] Grimm J.W., See R.E. (1998). Unique activation of extracellular striato-pallidal neurotransmitters in rats following acute risperidone. Brain Res..

[54] Xu S., Gullapalli R.P., Frost D.O. (2015). Olanzapine antipsychotic treatment of adolescent rats causes long term changes in glutamate and GABA levels in the nucleus accumbens. Schizophr. Res..

[55] Pan B., Lian J., Huang X.-F., Deng C. (2016). Aripiprazole Increases the PKA Signalling and Expression of the GABAA Receptor and CREB1 in the Nucleus Accumbens of Rats. J. Mol. Neurosci..

[56] Chadha R., Meador-Woodruff J.H. (2020). Downregulated AKT-mTOR signaling pathway proteins in dorsolateral prefrontal cortex in Schizophrenia. Neuropsychopharmacology.

[57] Dummler B., Hemmings B.A. (2007). Physiological roles of PKB/Akt isoforms in development and disease. Biochem. Soc. Trans..

[58] Matsuda S., Ikeda Y., Murakami M., Nakagawa Y., Tsuji A., Kitagishi Y. (2019). Roles of PI3K/AKT/GSK3 Pathway Involved in Psychiatric Illnesses. Diseases.

[59] Bitanihirwe B.K.Y., Weber L., Feldon J., Meyer U. (2010). Cognitive impairment following prenatal immune challenge in mice correlates with prefrontal cortical AKT1 deficiency. Int. J. Neuropsychopharmacol..

[60] Willi R., Harmeier A., Giovanoli S., Meyer U. (2013). Altered GSK3β signaling in an infection-based mouse model of developmental neuropsychiatric disease. Neuropharmacology.

[61] Bitanihirwe B.K.Y., Peleg-Raibstein D., Mouttet F., Feldon J., Meyer U. (2010). Late prenatal immune activation in mice leads to behavioral and neurochemical abnormalities relevant to the negative symptoms of schizophrenia. Neuropsychopharmacology.

[62] Baghel M.S., Singh B., Patro N., Khanna V.K., Patro I.K., Thakur M.K. (2019). Poly (I:C) Exposure in Early Life Alters Methylation of DNA and Acetylation of Histone at Synaptic Plasticity Gene Promoter in Developing Rat Brain Leading to Memory Impairment. Ann. Neurosci..

[63] Su Y., Liu X., Lian J., Deng C. (2020). Epigenetic histone modulations of PPARγ and related pathways contribute to olanzapine-induced metabolic disorders. Pharmacol. Res..

[64] Tang B., Jia H., Kast R.J., Thomas E.A. (2013). Epigenetic changes at gene promoters in response to immune activation in utero. Brain Behav. Immun..

